# Loss of HOXB3 correlates with the development of hormone receptor negative breast cancer

**DOI:** 10.7717/peerj.10421

**Published:** 2020-11-20

**Authors:** Lizhe Zhu, Shibo Yu, Siyuan Jiang, Guanqun Ge, Yu Yan, Yuhui Zhou, Ligang Niu, Jianjun He, Yu Ren, Bin Wang

**Affiliations:** Department of Breast Surgery, The First Affiliated Hospital of Xi’an Jiaotong University, Xi’an, China

**Keywords:** HOXB3, Hormone receptor negative, Breast cancer, Bioinformatic, Prognosis

## Abstract

**Background:**

The homeobox gene family, encoding a specific nuclear protein, is essential for embryonic development, differentiation, and homeostasis. The role of the HOXB3 protein varies in different tumors. This study aims to explore the role of the HOXB3 gene in breast cancer.

**Method:**

Differentially expressed genes were screened by analyzing metastatic breast cancer gene chip data from TCGA and GEO databases. The function of the selected HOXB3 gene was also analyzed in different databases and through molecular biology methods, such as qRT-PCR, western blot and IF to verify bioinformatics findings.

**Results:**

Both bioinformatics analyses and western blot showed that HOXB3 was lost in breast cancer compared to normal breast tissue. Survival analysis also showed that lower expression of HOXB3 was associated with poor prognosis. Bioinformatics analyses further showed that HOXB3 was positively correlated with hormone receptors. Metascape for GO analysis of GEO data provided possible mechanisms that HOXB3 could positively regulate cell adhesion, inhibit cell proliferation and activate immune response in breast cancer; moreover, GSEA included several cancer-associated pathways.

**Conclusion:**

In summary, HOXB3 expression was decreased in breast cancer, and it was associated with poor prognosis. It might become a new biomarker to predict prognosis of breast cancer.

## Introduction

The incidence of breast cancer among women in China has consistently increased in recent years. The annual incidence of breast cancer in women in China is approximately 208 000, and the total crude incidence rate is 32.43/100 000. This incidence accounts for 16.20% of all female cancer cases, the highest of all cancer incidences (Zeng et al., 2014). A recent study showed that the rapid growth rate in Chinese females has exceeded the world average growth rate of 2% (Shen & Sun, 2018). At the same time, breast cancer has become one of the leading causes of death among young women in China (National Bureau of Statistics of China, 2010). In the United States, breast cancer is the second most common cancer among women, after skin cancer, and it is also the second leading cause of cancer death, after lung cancer (DeSantis et al., 2017). Among women between the ages of 30 and 59, the most common cancer diagnosed is breast cancer, and among women under the age of 45, breast cancer is the leading cause of death. Breast cancer, as a malignant tumor with a complicated biological behavior in the female reproductive system, has seriously harmed women’s lives and health.

The development of genomics engineering has opened up new fields for studying the pathogenesis of breast cancer, especially for the genes involved in its growth pathways. Among them, the homeobox (HOX) regulatory gene family contains a homologous domain transcription factor that can encode a specific nuclear protein as a transcription factor, which is crucial for embryonic development, differentiation, and homeostasis. In recent years, many studies have shown that the imbalance of HOX genes is inextricably associated with the occurrence and invasion of various tumors. Laixi Bi et al. found that a miR-375-HOXB3-CDCA3/DNMT3B regulatory pathway, containing the HOXB3 gene, was involved in the development of acute myeloid leukemia (Bi et al., 2018). Yang et al. (2016) found that downregulation of miRNA-375 could inhibit the proliferation, migration and chemo-sensitivity of pancreatic cancer by combining with HOXB3. Chen et al. (2013) and others showed that HOXB3 could promote the progression of prostate cancer cells by activating CDCA3. Miller et al. (2018) and others demonstrated that HOXA4/HOXB3 gene expression could be used as a marker of recurrence, after primary cytoreductive surgery and first-line adjuvant chemotherapy, for high-grade serious ovarian cancer. Thus, the abnormal expression of the HOX gene family has been reported in a variety of malignant tumors with abnormal development, suggesting that changes in HOX genes might have important implications for tumorigenesis and its inhibition.

At present, there are few studies on the HOXB3 gene in breast cancer. Therefore, this study screened differentially expressed genes of breast cancer metastases by analyzing breast cancer metastasis-related gene chip data in databases, such as TCGA and the gene chip public database (GEO). Metascape, Breast Cancer Gene-Expression Miner and other software were used to analyze data. qRT-PCR, western blot, immunofluorescence and other molecular biology methods were applied to verify clinical findings and analyze their association with breast cancer immunophenotyping and prognostic values. Thus, our data provided insights and ideas for further exploring molecular metastasis of breast cancer.

## Materials and Methods

### GEPIA

GEPIA (Gene Expression Profiling Interactive Analysis, http://gepia.cancer-pku.cn/) (Tang et al., 2017) is a web tool that was used for cancer and normal gene expression profiling, as well as but also for interactive analyses. This web tool can analyze RNA sequencing expression data from approximately 9736 tumors and 8587 normal samples. All samples are excavated from TCGA and GTEx projects using a standard processing pipeline. It provides different kinds of functions, including tumor vs normal differential expression analysis, patient survival analysis, similar gene detection, etc. The differential expression of HOXB3 in normal breast tissue and breast cancer was detected by GEPIA using one-way ANOVA. A log *P*-value < 0.01 was considered statistically significant.

### Kaplan–Meier Plotter database

The Kaplan–Meier Plotter (Lánczky et al., 2016; Györffy et al., 2010) is a tool that is based on meta-analysis biomarker assessment. This web tool can assess the survival of patients across multiple tumors, such as breast, ovarian, lung, etc. The gene expression data and relapse free survival information are downloaded from TCGA, GEO and EGA. Handled by a PostgreSQL server, this database is able to integrate clinical data and gene expression data simultaneously. We used this database to analyze the prognostic value of HOXB3 mRNA expression in all breast cancers. A log *P*-value < 0.01 was considered statistically significant.

### Breast Cancer Gene-Expression Miner (bc-GenExMiner) v4.3

Bc-GenExMiner v4.3 (Jézéquel et al., 2012; Jézéquel et al., 2013) is a statistical mining tool that includes a large number of published, annotated breast cancer mRNA data. It can perform the statistical analyses for prognosis, expression and correlation. The correlation between HOXB3 and ESR1, ESR2, PGR, ERBB2 and MKI67 were analyzed by using Bc-GenExMiner v4.3. The relationship between HOXB3 and the clinicopathologic parameters (ER, PR, HER2, SBR, molecular subtypes) of breast cancer were also analyzed on this online tool.

### GEO database and Metascape

We downloaded the original dataset for comparing the gene expression profile between breast cancer and normal breast tissue from the NCBI GEO database (accession number: GSE27447). Later, we used GEO2R and Metascape to perform GO and KEGG pathway analyses on the target gene HOXB3. Metascape (http://metascape.org) (Zhou et al., 2019) is a gene annotation and analysis resource that was used for gene enrichment analysis, and it also found relevant signaling pathways.

### TIMER and R&D Systems immune cell markers

The TIMER web server is a comprehensive resource for the systematic analysis of immune infiltrates across diverse cancer types (Li et al., 2017; Li et al., 2016). We selected the representative gene markers of immune cells noted on the R&D Systems website (https://www.rndsystems.com/cn/resources/cell-markers/immune-cells). Different genes represented different immune cells; we used TIMER to analyze the correlation between HOXB3 expression and all these marker genes. In addition, we explored the association of immune infiltration levels among cancers with different somatic copy number alterations (SCNAs) affecting HOXB3 expression. The infiltration level for each SCNA category was compared with that for normal breast tissue using a two-sided Wilcoxon rank-sum test.

### R software

The relationship between HOXB3 with M stages and clinical stages, and the preparation documents of the GSEA were all performed with R software (version 4.0.0; http://www.r-project.org). The packages in R that we used are as follows: “ggstatsplot”, “ggpubr”, “plyr”, “ggplot2”, “grid”, “gridExtra”, “survival” and “survminer”.

### Cell culture and reagents

Human breast cancer cells MDA-MB-231, MCF-7, T47D and SUM159 (obtained from American Type Culture Collection and preserved in our lab) were maintained in L15, DMEM High Glucose, RPMI 1640 medium and DMEM/F12 (Gibco; Thermo Fisher Scientific, Inc., Waltham, MA, USA), respectively and were supplied with 10% fetal bovine serum (FBS; Gibco; Thermo Fisher Scientific, Inc.) at 37 °C with 5% CO2. The MCF-10A cells were cultured in DMEM/F12 (1:1) (Gibco; Thermo Fisher Scientific, Inc., Waltham, MA, USA) that was supplemented with 100 ng/ml cholera toxin (Sigma, St. Louis, MO, USA), 20 ng/ml epidermal growth factor (EGF) (Thermo Fisher Scientific, Inc., Waltham, MA, USA), 10 µg/ml insulin, 500 ng/ml hydrocortisone and 5% heat-inactivated horse serum (all from Sigma) at 37 °C with 5% CO2. Old cell culture medium was replaced with fresh medium every other day.

### Real-time quantitative polymerase chain reaction (qRT-PCR)

Total RNA was extracted with the RNeasy mini kit (Qiagen, Valencia, CA) according to the manufacturer’s protocol. Concentration and purity of all RNA samples were determined at an absorbance ratio of 260/280 nm. A total of 1 µg RNA was reverse-transcribed using iScriptTM cDNA Synthesis kit from Bio-Rad (Hercules, CA, USA). Real-time PCR analysis was set up with the SYBR Green qPCR Supermix kit (Invitrogen, Carlsbad, CA) and carried out in the iCycler thermal cycler. The relative level of mRNA expression of a gene was determined by normalizing with GAPDH. Primers for HOXB3: forward-5′TGCTGCTGGGAGACTCGTAA 3′; reverse-5′GCATCCCCTTGCAGCTAAAC 3′; GAPDH: forward-5′AAGGCTGTGGGCAAGGTCATC 3′; reverse-5′GCGTCAAAGGTGG AGGAGTGG 3′.

### Western blot

The expression level of HOXB3 in different molecular subtypes of breast tissues was analyzed by western blot. Human breast tissues were minced and transferred into a homogenizer, and later lysed in RIPA buffer, containing phosphatase and a protease inhibitor, by using an ultrasonic crusher (SONICS and MATERIALS INC. USA). Then, the breast tissue homogenate was kept on ice for 20 min and transferred to a centrifuge tube for centrifugation at 4 °C, 20 min. The breast tissue proteins from the upper-part of the supernatant were collected and detected using a BCA Protein Assay Kit (Pierce; Thermo Fisher Scientific, Inc.). Then, 30 µg of protein was separated on 10% SDS-PAGE gels and transferred to polyvinylidene fluoride (PVDF) membranes (Merck Millipore, Billerica, MA, USA). The membranes were blocked with 5% nonfat milk in TBST to prevent nonspecific binding and subsequently incubated with primary antibody (HOXB3: cat no. ITA9318; G-Biosciences, Inc.; dilution, 1:2,000; GAPDH: cat no. 5174; Cell Signaling Technology, Inc.; dilution, 1:1,000) overnight at 4 °C. All samples were incubated with anti-horseradish peroxidase-linked IgG secondary antibody (cat no. 7074; Cell Signaling Technology, Inc.; dilution, 1:2,000) at room temperature for 2 h and detected via chemiluminescence detection system (version 3.0; Bio-Rad Laboratories, Inc., Hercules, CA, USA).

### Immunofluorescence

Cells were washed with PBS and fixed with 4% paraformaldehyde. Subsequently, 0.5% Triton X-100 was used to perforate cells, and 5% bovine serum albumin (BSA) was used to block cells. Cells were then incubated with primary antibody (HOXB3: cat no. ITA9318; G-Biosciences, Inc.) at 4 °C overnight and Alexa Fluor 647-conjugated secondary antibodies, followed by nuclear staining with 5 ug/ml 4′,6-diamidino-2-phenylindole (DAPI, Invitrogen). Signals were examined using a fluorescence microscope.

### Clinical specimens, ethics approval and consent to participate

All the breast cancer samples were obtained from the department of pathology of the First Affiliated Hospital of Xi’an Jiaotong University. All clinical specimens came from patients within 30 min after surgery. Tumor tissue and adjacent normal tissue were confirmed by a pathologist and frozen with liquid nitrogen to prevent protein degradation. The human breast cancer tissues used in this study have received consent from the Ethics Committee of the First Affiliated Hospital of Xi’an Jiaotong University. Additionally, written informed consent from all participants was received. The other data in this study were from online databases, which do not require ethical approval.

## Results

### Decreased expression of HOXB3 in breast cancer patients

**Figure 1:**
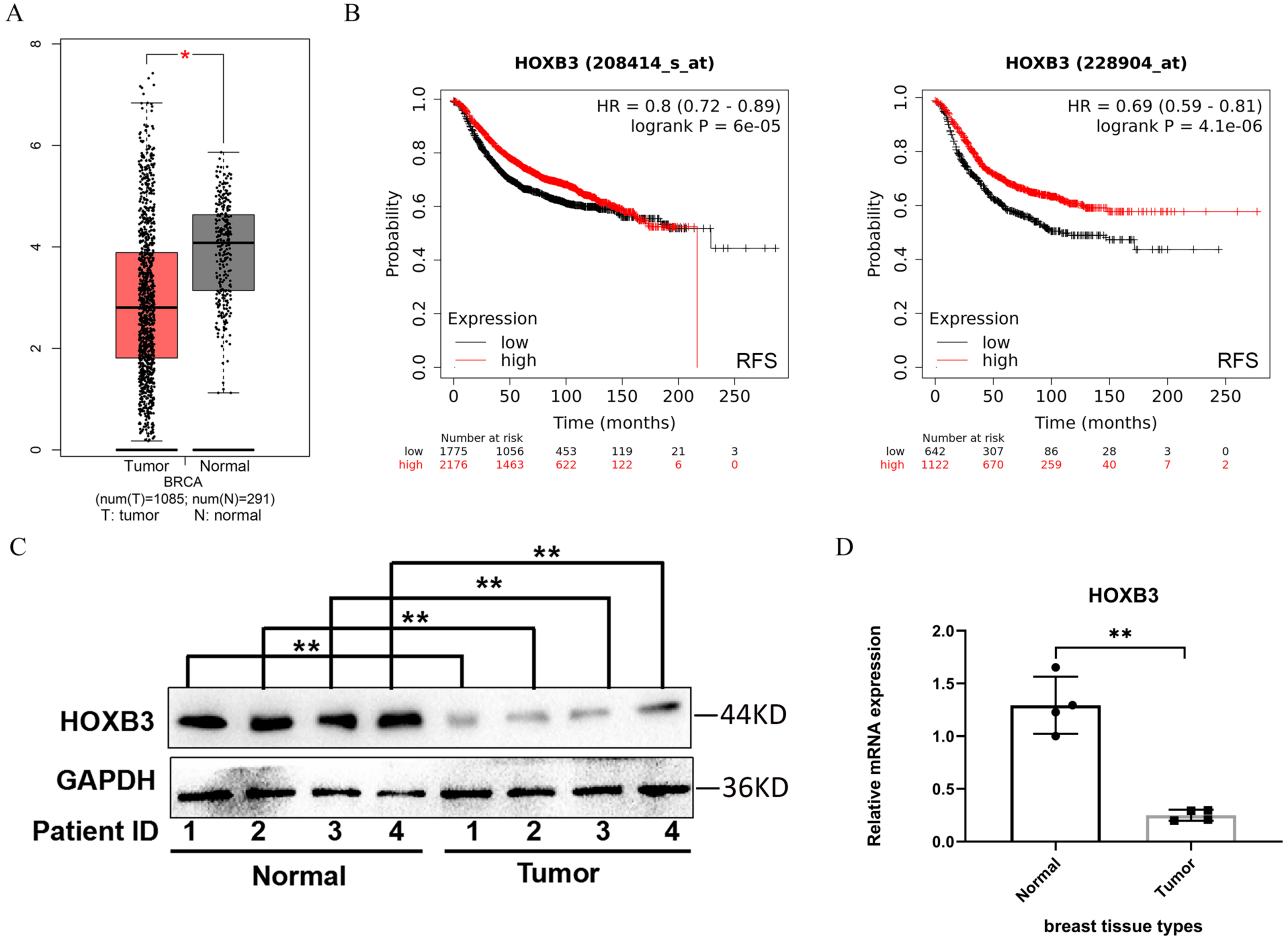
HOXB3 expression in breast cancer patients compared with healthy controls. (A) Box plot of HOXB3 expression in breast cancer patients compared with healthy controls in the GEPIA database (we used log2(TPM + 1) as the *y*-axis. TPM: Transcripts Per Million). (B) Survival analyses of HOXB3 in breast cancer (RFS in Kaplan–Meier Plotter, the software auto selects the best cutoff to split patients). (C) Western blot of HOXB3 expression in human breast tissues compared with normal tissue. (D) The relative protein expression of HOXB3 (GAPDH normalized) from the western blot (the protein qualification of Figure 1C). *P* < 0.05 was considered statistically significant. (“*” represents *P* < 0.05, “**” represents *P* < 0.01).

HOXB3 expression was analyzed in breast cancer patients compared with healthy breast tissue in the GEPIA database. Figure 1A shows that the expression of the HOXB3 gene in breast cancer patients was significantly lower than in normal tissue, and the difference was statistically significant. The Kaplan–Meier Plotter was used to examine the prognostic values of HOXB3 mRNA expression level in all breast cancers (Fig. 1B). It revealed that low mRNA expression of HOXB3 was associated with a worse prognosis of RFS (Left, HR = 0.8, 95% CI [0.72–0.89], P = 6e−05; Right, HR = 0.69, 95% CI [0.59–0.81], *P* = 4.1e−06). The results were in line with the data showing that lower expression of HOXB3 existed in breast cancer patients. In addition, survival analyses of HOXB3 in breast cancer can be seen in Table S1. We further tested the HOXB3 gene expression in mRNA level and protein level which extracted from human breast tissues. Figures. 1C and 1D showed that HOXB3 was downregulated both in protein and mRNA levels in breast cancer tissues compared with normal tissue. These data suggested that HOXB3 might be decreased in breast cancer patients and even play critical roles in breast cancer cell carcinogenesis.

### The relationship between HOXB3, hormone receptor-related genes and IHC related genes

**Figure 2:**
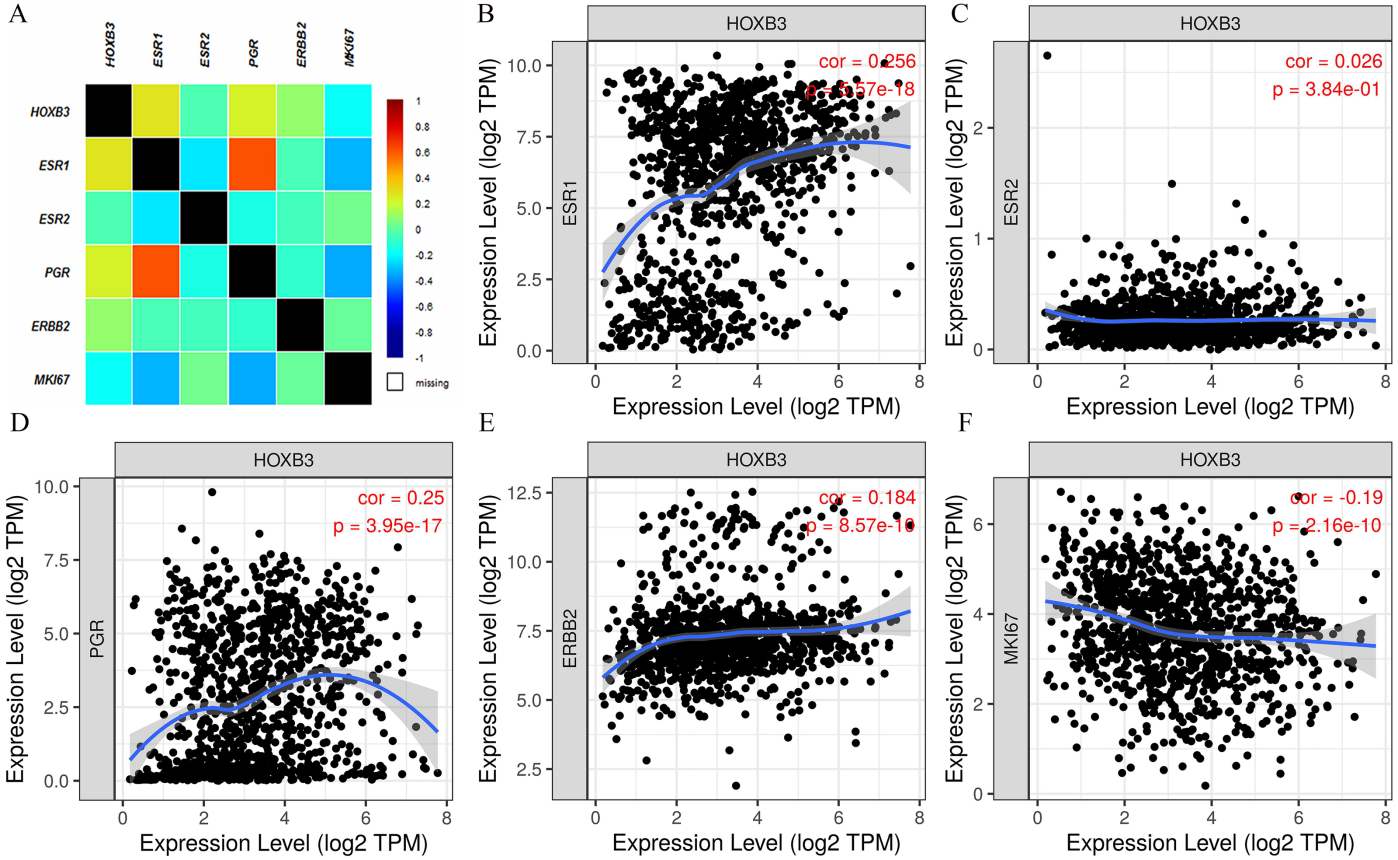
The correlation between HOXB3 and clinicopathologic parameters of breast cancer. (A) The total correlation heatmap of HOXB3 expression with clinicopathologic parameters (Different colors represented different degrees of correlation from −1 to 1. Blue represents the highest degree of negative correlation, and red represents the highest degree of positive correlation). (B) The correlation between HOXB3 and ESR1. (C) The correlation between HOXB3 and ESR2. (D) The correlation between HOXB3 and PGR. (E) The correlation between HOXB3 and ERBB2. (F) The correlation between HOXB3 and MKI67. *P* < 0.05 was considered statistically significant.

**Figure 3:**
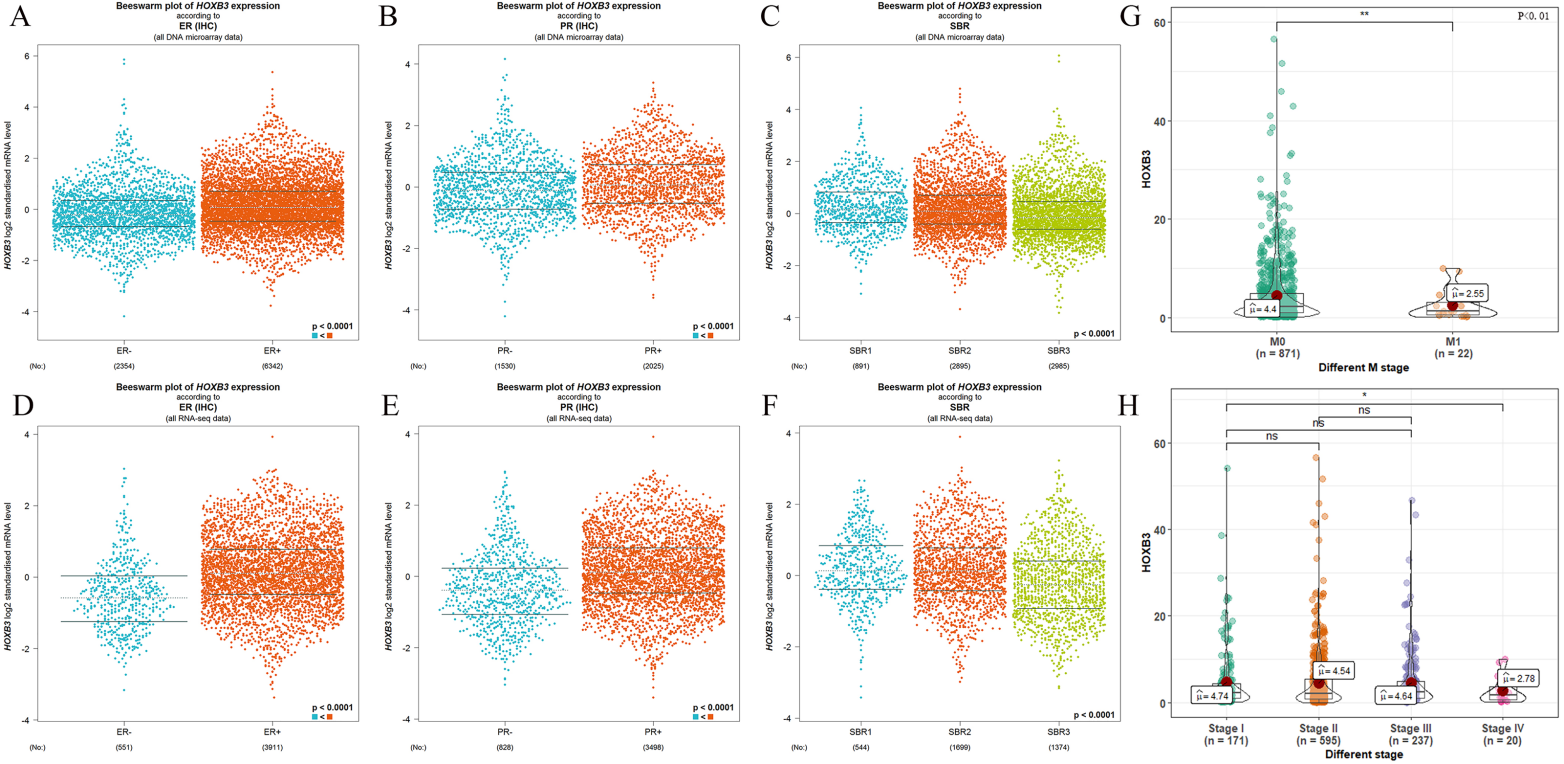
The relationship between HOXB3 and ER, PR, SBR criteria, M stages and clinical stages. (A, B, C) Box plots of HOXB3 expression according to ER, PR and SBR (from DNA microarray). (D, E, F) Box plots of HOXB3 expression according to ER, PR and SBR (from RNA-sequencing). (G) The relationship between HOXB3 and M stages. (H) The relationship between HOXB3 and clinical stages. Global significant differences between groups were assessed by Welch’s test, and *P* < 0.05 was considered statistically significant.

It is well documented in current clinical guidelines that the classic treatment plan for breast cancer was to first classify and then treat. The specific molecular classification was mainly based on the expression of ER and PR. Clinical practices had also proved that the expression levels of ER and PR played an important role in judging the degree of malignancy of breast cancer and affected the decision of treatment plan. Figure 2 shows that HOXB3 was highly correlated with hormone receptor-related genes, such as ESR1 and PGR. Genes for IHC classification, such as ERBB2 and MKI67, were also shown the to correlate ion with HOXB3. Bc-GenExMiner v4.3 and TIMER were used to analyze the relationship between HOXB3 and those clinicopathologic parameters of breast cancer. Results showed that HOXB3 was positively correlated with ESR1, PGR and ERBB2 (ESR1: *r* = 0.256, *P* < 0.0001; PGR: *r* = 0.25, *P* < 0.0001; ERBB2: *r* = 0.184, *P* < 0.0001), whereas it was negatively correlated with proliferative maker Ki67 (MKI67: r=-0.19, *P* < 0.0001) (Fig. 2). Figures 3A–3C and 3D–3F also showed that higher mRNA expression of HOXB3 was associated with positive expression of hormone receptors (ER and PR). Furthermore, for the Scarff-Bloom-Richardson (SBR) grade criterion, shown in Figs. 3C and 3F, a higher mRNA level of HOXB3 was associated with a lower SBR grade in both cDNA microarrays and RNA-sequencing data. All of the pairwise comparisons in the SBR criteria were statistically significant (*P* < 0.01). We also used R software to analyze the 1066 breast cancer patients in TCGA. Figure 3G shows that lower HOXB3 expression corresponded to distant metastasis of breast cancer. Additionally, the HOXB3 expression in stage IV breast cancer patients was significantly lower than stage I (Fig. 3H).

### HOXB3 mRNA expression in different molecular subtypes of breast cancer

**Figure 4:**
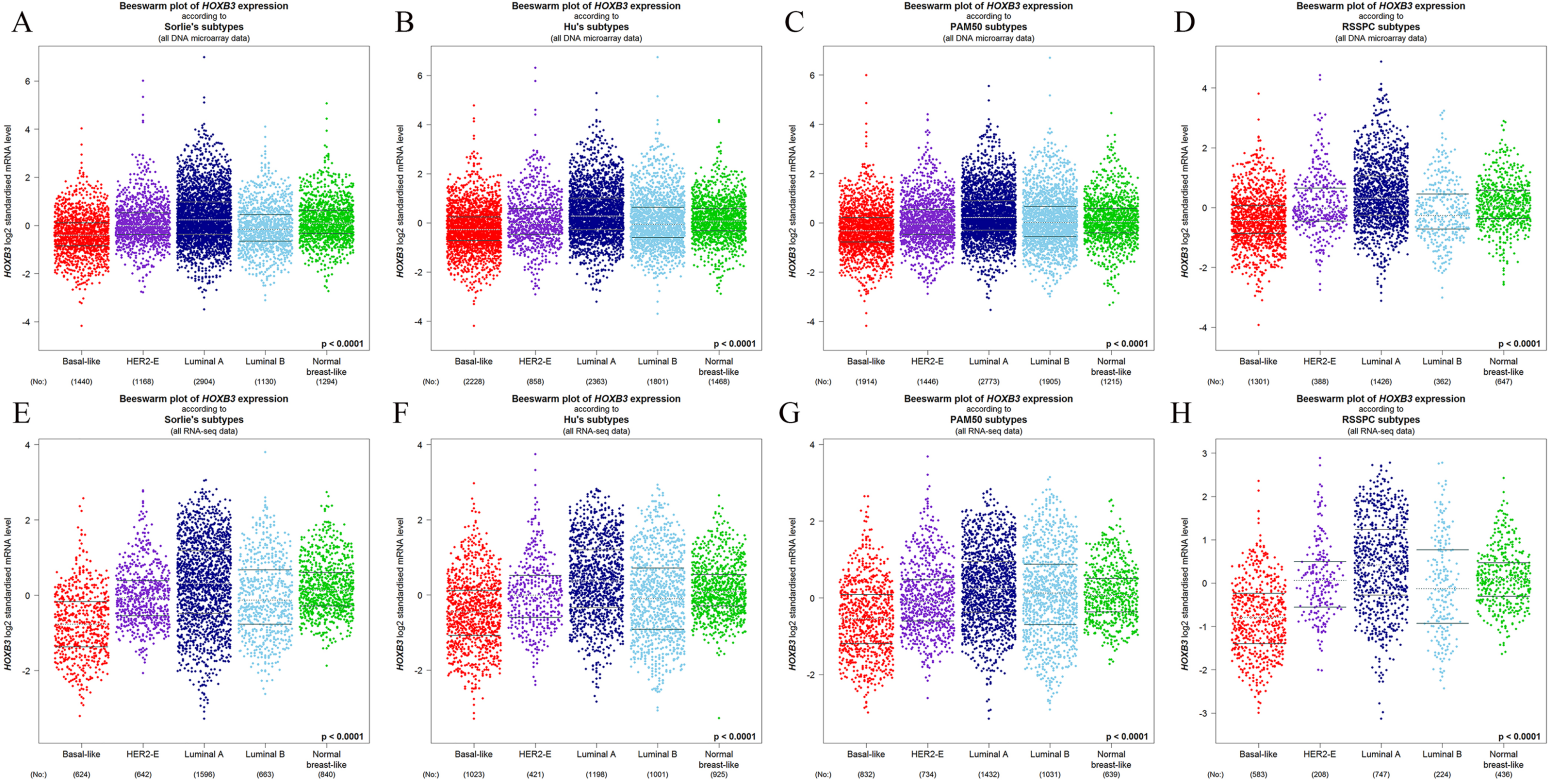
The relationship between HOXB3 and different molecular subtypes of breast cancer. (A–D) Box plots of HOXB3 expression according to different molecular subtypes (from DNA microarrays). (E–H) Box plots of HOXB3 expression according to different molecular subtypes (from RNA-sequencing). Global significant differences between groups were assessed by Welch’s test, and *P* < 0.05 was considered statistically significant.

**Figure 5:**
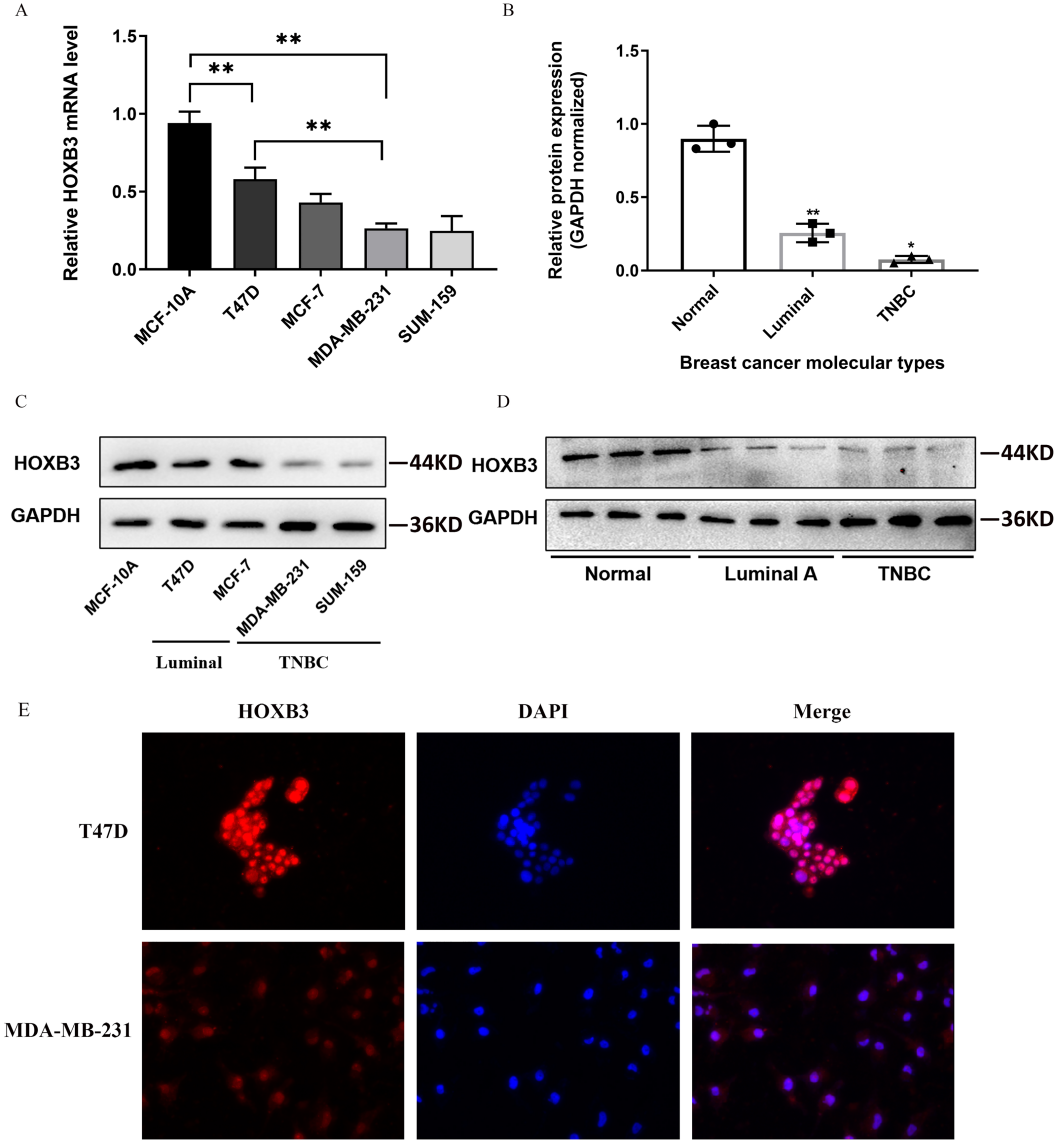
HOXB3 expression in breast cancer cell lines and breast cancer tissues. (A) The relative HOXB3 mRNA level of MCF-10A, T47D, MCF-7, MDA-MB-231 and SUM-159 by qRT-PCR. (B) The relative protein expression of HOXB3 (GAPDH normalized) by western blot (the protein qualification of Figure 5D). (C) western blot of HOXB3 expression in breast cancer cell lines including MCF-10A, T47D, MCF-7, MDA-MB-231 and SUM-159. (D) Western blot of HOXB3 expression in normal breast tissues, luminal breast cancer and TNBC. (E) Immunofluorescence of HOXB3 expression in T47D and MDA-MB-231. *P* < 0.05 was considered statistically significant. (“*” represents *P* < 0.05, “**” represents *P* < 0.01).

Figure 4 shows that both at the DNA microarray and RNA-sequencing levels, higher mRNA expression of HOXB3 was correlated with normal breast-like tissues or luminal subtypes of breast cancer. A relatively lower mRNA level of HOXB3 was correlated with HER2 or basal-like breast cancer. We further detected specific gene expression with molecular biology methods. By qRT-PCR, we confirmed that HOXB3 had the highest expression in the immortalized breast epithelial cell line MCF-10A, whereas expression was lower in the luminal breast cancer cell lines T47D and MCF-7 and the lowest in the triple negative breast cancer (TNBC) cell lines MDA-MB-231 and SUM-159 (Fig. 5A). Moreover, western blot showed that HOXB3 was highly expressed in normal breast tissues, moderately expressed in luminal breast cancer, and lowly expressed in TNBC (Figs. 5B and 5D). Similar results were seen in the different types of breast cancer cell lines (Fig. 5C). Immunofluorescence showed that the HOXB3 gene was localized to the nucleus. Additionally, the fluorescence intensity was strong in the luminal breast cancer cell line T47D, and weak in the TNBC cell line MDA-MB-231 (Fig. 5D). It was suggested that the HOXB3 gene might have lower expression in aggressive breast cancer subtypes.

**Figure 6:**
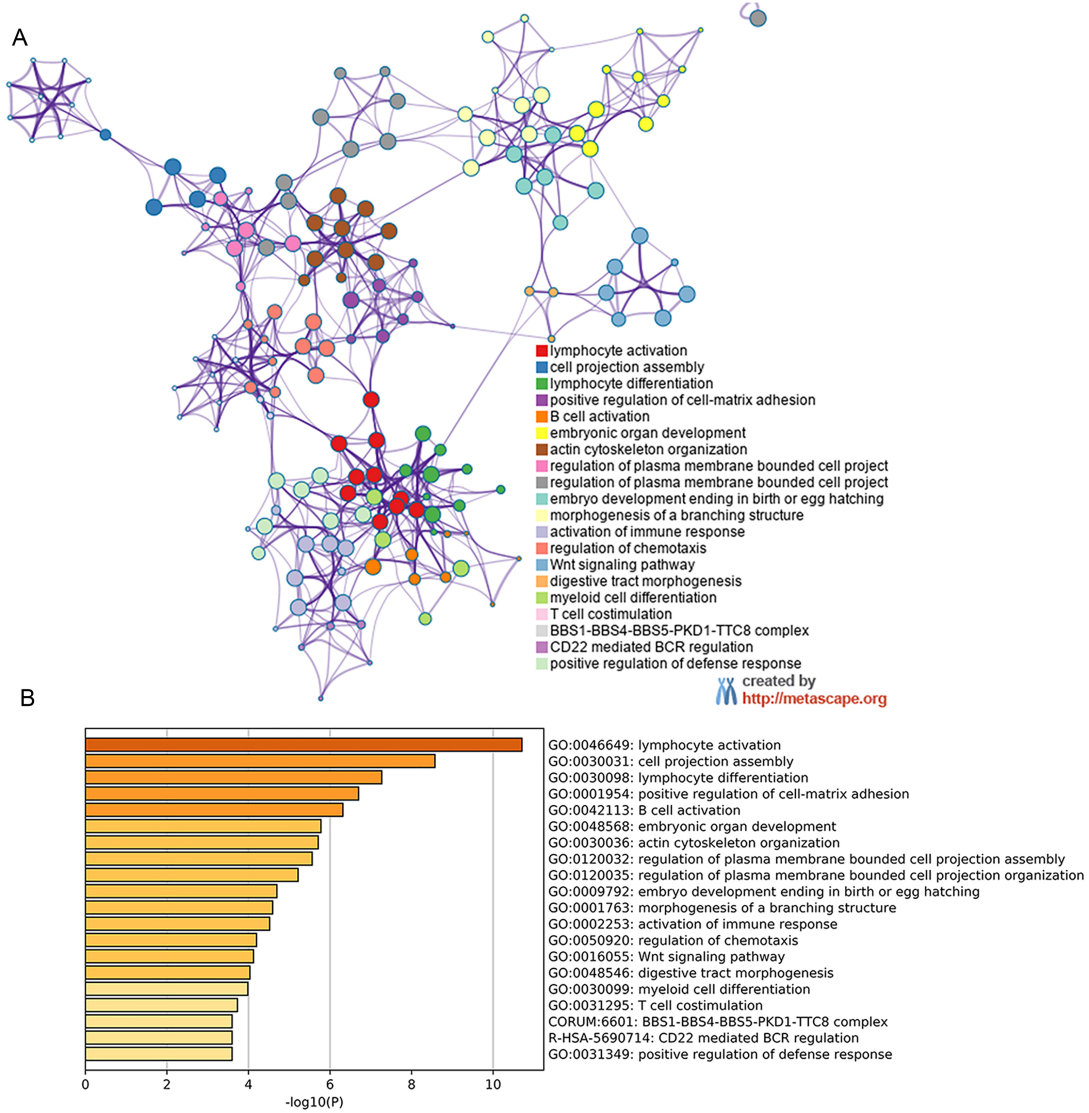
Metascape for GO analysis of possible mechanism of HOXB3 in breast cancer. A discrete color scale was used to represent statistical significance, and the abscissa represents the -log10(P) value of GO analysis.

**Table 1:** The correlation between HOXB3 expression and immune cells related gene markers in breast cancer (TIMER database).

Cell type	Gene marker	Cor	*P*
B cell	CD19	−0.047	0.689
	CD20	−0.538	^***^
	CD38	−0.555	^***^
CD8+ T Cell	CD8A	−0.565	^***^
	CD8B	−0.618	^***^
Tfh	CXCR5	−0.216	0.064
	ICOS	−0.600	^***^
	BCL-6	0.250	^*^
Th1	IL12RB2	−0.208	0.075
	WSX-1	−0.191	0.104
	T-BET	−0.617	^***^
Th2	CCR3	−0.365	^**^
	STAT6	0.147	0.213
	GATA-3	0.180	0.124
Th9	TGFBR2	−0.344	^**^
	IRF4	−0.658	^***^
	PU.1	−0.603	^***^
Th17	IL-21R	−0.204	0.082
	IL-23R	0.199	0.088
	STAT3	0.062	0.598
Th22	CCR10	0.006	0.957
	AHR	−0.071	0.546
Treg	FOXP3	0.009	0.937
	CCR8	−0.330	^**^
	CD25	−0.509	^***^
T cell exhaustion	PD-1	−0.581	^***^
	CTLA4	−0.504	^***^
Macrophage	CD68	−0.529	^***^
	CD11b	−0.594	^***^
M1	NOS2	−0.154	0.192
	ROS	−0.274	^*^
M2	ARG1	0.042	0.723
	MRC1	−0.512	^***^
TAM	HLA-G	−0.130	0.268
	CD80	−0.241	^*^
	CD86	−0.531	^***^
Monocyte	CD14	−0.611	^***^
	CD16	−0.503	^***^
NK	XCL1	−0.523	^***^
	KIR3DL1	−0.302	^**^
	CD7	−0.665	^***^
Neutrophil	CD15	0.027	0.816
	MPO	−0.243	^*^
DC	CD1C	−0.580	^***^
	CD141	−0.442	^***^

**Notes.**

* *P* < 0.05.

** *P* < 0.01.

*** *P* < 0.001.

Abbreviations Tfh follicular helper T cell Th T helper cell Treg regulatory T cell TAM tumor associated macrophage NK natural killer cell DC dendritic cell None correlation without adjustment Purity correlation adjusted for tumor purity Cor R value of Spearman’s correlation

### Metascape for GO analysis of possible mechanism of HOXB3 in breast cancer

By analyzing the NCBI GEO database, we found the differentially expressed gene HOXB3. Later, GEO2R and Metascape was used to perform GO and KEGG pathway analysis on our target gene HOXB3. The bioinformatic analysis revealed the popular signaling pathways related to HOXB3. As shown in Fig. 6, the related signaling pathways are involved in positive regulation of cell matrix adhesion, actin cytoskeletal remodeling, chemotaxis regulation, classical Wnt signaling and activation of the immune response. Those possible mechanisms suggested that HOXB3 could positively regulate cellular adhesion, inhibit cell proliferation and activate the immune response in breast cancer, as well as made us consider that HOXB3 might cause malignant cell transformation through the above pathways. In recent years, immunotherapy has been a hot topic in cancer research, therefore we investigated the relationship between HOXB3 expression and representative immune markers of several immune cells (Table 1). The results showed that HOXB3 was significantly correlated with B cell, CD8+ T cell, Tfh, Th1, Th2, Th9, Treg, T cell exhaustion, macrophage, M1, M2, TAM, Monocyte, NK, neutrophil and DC specific markers. Fig. S1 showed that the HOXB3 gene was frequently altered in breast cancer and the association between HOXB3 copy number variations and immune infiltrates in breast cancer was statistically significant. These findings further verified that HOXB3 expression might play an important role in immune infiltration in breast cancer.

### Clinical correlation and multiple GSEA of possible KEGG pathways of HOXB3 in breast cancer

**Figure 7:**
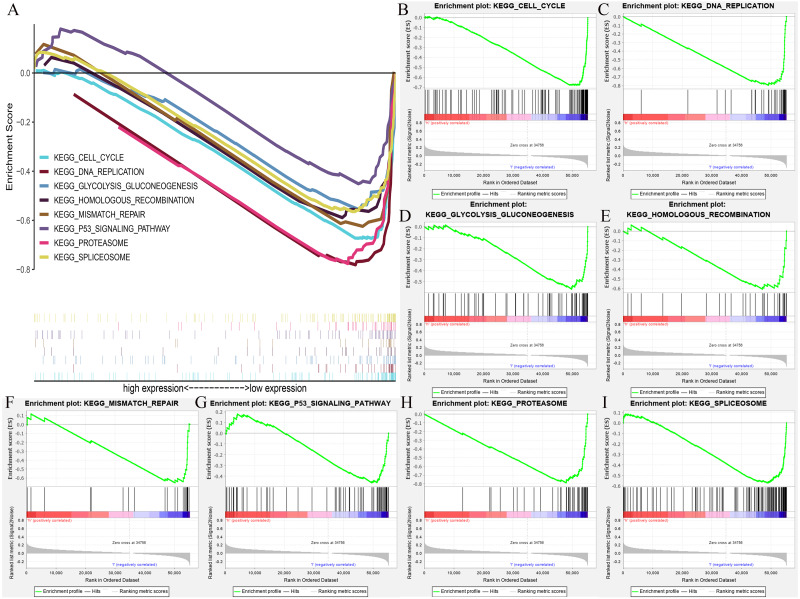
The GSEA (Gene set enrichment analysis) of possible pathways of HOXB3 in breast cancer. (A) The summary of all significant GSEA pathways of HOXB3 in breast cancer. (B) The enrichment plot of cell cycle. (C) The enrichment plot of DNA replication. (D) The enrichment plot of glycolysis and gluconeogenesis. (E) The enrichment plot of homologous recombination. (F) The enrichment plot of mismatch repair. (G) The enrichment plot of the P53 signaling pathway. (H) The enrichment plot of the proteasome pathway. (I) The enrichment plot of the spliceosome pathway.

We further extracted the clinical data of breast cancer in TCGA for univariate and multivariate COX regression analyses (Table S2). The results showed that HOXB3 might be an independent risk prognostic factor for breast cancer (*P* = 0.03). Fig. S2 showed the results of our multifactor analysis more visually. To further predict the possible KEGG pathways based on the TCGA data, we did GSEA using R software and GSEA 4.0.3. The results showed that high HOXB3 expression might inhibit the following pathways: cell cycle, DNA replication, glycolysis gluconeogenesis, homologous recombination, mismatch repair, P53 signaling pathway, proteasome and spliceosome (Fig. 7). All the cancer-related KEGG pathways surrounding HOXB3 indicated that it might play an important role in breast cancer.

## Discussion

Some researchers believe that the occurrence of tumors mimics the process of early embryonic development and aberration (Shah & Sukumar, 2010). Research on embryonic development-related genes and tumorigenesis has become another research hotspot in molecular oncology, where homeobox gene, HOXB3, is one of them (Daftary & Taylor, 2006; Chen & Capecchi, 1999; Kar, Tyrer & Li, 2015). HOXB3 has different effects in promoting or suppressing cancer in different types of tumors. Fu et al. (2017) and others found that HOXB3 is a target of miR-375 in MCF-7 cells. Moreover, HOXB3 could not only significantly promote EMT in MCF-7 cells but also promote the formation of a CSC phenotype and tamoxifen resistance. Li, Zhu & Chen (2012) found that miR-7 and miR-218 can regulate breast cancer suppressor genes RASSF1A and Claudin-6 through targeting HOXB3. Although both studies considered HOXB3 to be an oncogene, they were all performed in a cellular model. In fact, the cellular model had great limitations and was affected by culture conditions that are unable to reflect the real situation of breast cancer patients. Our study analyzed a great number of data, the international common databases (TCGA and GEO) and verified patient specimens by molecular biology methods. The mRNA and protein expression of HOXB3 was significantly lower in breast cancer tissues compared to normal tissue. This result was consistent with Svingen’s team (Svingen & Tonissen, 2003). Their team verified the expression in normal breast tissue, the normal breast cell line SVCT, breast cancer cell line MCF-7 and MDA-MB-231. They found that the expression of HOXB3 in breast cancer cells was much lower than that found in normal breast tissue and the normal breast cell line SVCT. Moreover, the HOXB3 expression in the melanoma cell lines MM96L, MM418c1 and MM48C5 was also much lower than that of human skin fibroblast HSF cells. As we showed in supplementary Fig. 3, HOXB3 was reduced to varying degrees in the expression profiles of multiple cancer types. This indicated that the loss of HOXB3 was prevalent in many cancers. HOXB3 might be a tumor suppressor gene in these specific cancers, including in breast cancer. In particular, HOXB3 was low expressed in breast cancer and melanoma. Anna A Brożyna et al. reported that progression of melanoma was clearly linked with defects in local vitamin D signaling (Brozyna et al., 2019; Brozyna, Hoffman & Slominski, 2020). Welsh (2018) also showed that the vitamin D deficiency is common in breast cancer patients and some evidence suggests that low vitamin D status enhances the risk for disease development or progression. Numerous anticancer properties of vitamin D have been proposed, with diverse effects on cancer development and progression, thus understanding dysregulated vitamin D metabolism and function in cancer will be critical for the development of promising new strategies for successful vitamin D-based cancer therapy (Jeon & Shin, 2018). Therefore, whether the loss of HOXB3 expression in breast cancer was mediated by the vitamin D signaling pathway and thus affected the occurrence and progression of breast cancer was worthy of further research.

Since the expression of HOXB3 was lost in breast cancer, it was unknown whether low expression of HOXB3 indicated a poor prognosis. Therefore, we verified the survival curve and found that low expression of the HOXB3 gene was associated with poor prognosis in breast cancer patients. To further predict the underlying mechanisms of HOXB3 function in breast cancer, we conducted two enrichment analyses. In Fig. 6, we used a TNBC dataset from the GEO database to analyze the signaling pathways that were enriched by genes that were highly related to the HOXB3 gene. In Fig. 7, we used breast cancer data from TCGA to carry out KEGG pathway enrichment analysis in GSEA. Through the enrichment analysis of multiple pathways, we screened for possible mechanisms for the HOXB3 gene to play a role in breast tumors. Enrichment of pathways through bio-informatics analysis of big data provided preliminary understanding of the gene under study and also a direction for our follow-up research to investigate the specific mechanism of this gene in breast cancer. Of the multiple pathways, we were interested in the positive regulation of cell–matrix adhesion, actin cytoskeleton organization, activation of immune response, Wnt signaling, cell cycle, and P53 signaling. For example, matrix adhesion is an important interdependent cell process in cell physiology and in cancer. The ability of cancer cells to adhere to their surrounding environment was the focus in the discovery of front-rear cell polarity driving cancer cell migration (Tan et al., 2019). In addition to cell–matrix interactions, the control of cytoskeleton dynamics are also important signals in cancer invasion and oncogene-mediated disruption of stress fibers. In addition, associated adhesive structures are known to have a critical role in pathways that increase motility and invasiveness of tumor cells, thereby facilitating metastasis. Changes in the organization of actin filaments are highly correlated with anchorage-independent growth, tumorigenicity and invasion, suggesting a fundamental role for actin fibers in cell growth control (Choi & Helfman, 2014). Wnt pathway and immune activation are also known to play multiple roles in tumor progression. Taken together, these are important pathways in oncology research, which meant that HOXB3 might play an important role in the progression of breast cancer. Consistent with the above results, TCGA data also suggested a negative correlation between HOXB3 and the cell proliferation marker Ki67. Therefore, we suspected that the absence of HOXB3 might lead to malignant transformation through the above pathways.

The molecular subtypes of breast cancer are an important variable in breast cancer research. Different molecular subtypes of breast cancer showed different malignant behaviors and required different treatments. Our study also analyzed HOXB3 in different subtypes of breast cancer. Our results suggested that there was a significant positive correlation between HOXB3 and hormone receptors ER and PR, but no significant correlation with HER2 expression. Clinically, hormone receptor-negative subtype indicates higher malignancy and worse prognosis. In our study, lower expression of hormone receptor was accompanied with lower expression of HOXB3. This principle was consistent with the findings of lower malignancy correlated with lower expression of HOXB3. Pathological evaluation of breast cancer is not only molecular classification but also SBR pathological grade. The higher the grade, the higher the malignancy. Consistently, the higher SBR pathological grade was accompanied with lower HOXB3 expression. These results all prove that HOXB3 may be a tumor suppressor gene, which is consistent with previous results. There are also some limitations of our study. Due to the improvement of breast cancer diagnosis and treatment level, most triple-negative breast cancers now use neoadjuvant chemotherapy, it is difficult to collect a large number of specimens of triple-negative breast cancer as before. we will try to collect tissue samples for protein extraction and immunohistochemistry to verify the expression of HOXB3 in the further mechanism research.

## Conclusions

In summary, we found that HOXB3 expression was decreased in breast cancer through data analysis and molecular biology experiments. This was especially the case in hormone receptor-negative breast cancer, where low expression of HOXB3 was associated with poor prognosis. Identifying people at risk with molecular characteristics may help to develop active surveillance, adjuvant therapies, clinical trial considerations, or alternative treatment intervention decisions for high-risk patients. Whether HOXB3 expression can be used as a prognostic biomarker for breast cancer patients needs further validation in prospective studies.

##  Supplemental Information

10.7717/peerj.10421/supp-1 Supplemental Information 1 The association between HOXB3 copy number variations and immune infiltrates (A) BRCA (breast invasive carcinoma), (B) BRCA-Basal (breast carcinoma-basal), (C) BRCA-Luminal (breast cancer-luminal), (D) BRCA-Her2 (breast cancer-her2).

10.7717/peerj.10421/supp-2 Supplemental Information 2 The forest plot of multivariate analysis of the correlation of HOXB3 expression with OS among breast cancer patients

10.7717/peerj.10421/supp-3 Supplemental Information 3 HOXB3 expression in different cancer types in GEPIA

10.7717/peerj.10421/supp-4 Supplemental Information 4 Survival analyses of HOXB3 in breast cancer Kaplan-Meier Plotter auto select best cutoff to split patients (all possible cutoff values between the lower and upper quartiles are computed, and the best performing threshold is used as a threshold). Bold values indicated P¡0.05. N represents number of included patients. RFS, recurrence free survival; OS, overall survival; DMFS, distant metastasis free survival; PPS, post progression survival; HR, hazard ratio; CI, confidence interval.

10.7717/peerj.10421/supp-5 Supplemental Information 5 Univariate analysis and multivariate analysis of the correlation of HOXB3 expression with OS among breast cancer patients Bold values indicated P¡0.05. HR, hazard ratio; CI, confidence interval.

10.7717/peerj.10421/supp-6 Supplemental Information 6 PCR raw materials

10.7717/peerj.10421/supp-7 Supplemental Information 7 Western blots

## Additional Information and Declarations

### Data Availability

The following information was supplied regarding data availability: The dataset for this study are available at GEO: GSE27447 and uncropped western blots and qRT-PCR raw data are available in the Supplemental Files. We also used: - GEPIA: the box plot in “Expression DIY” of “Expression Analysis”, enter the “HOXB3” in “gene” module and get the box plot of the differential expression of HOXB3 in normal breast tissue and breast cancer. - Kaplan-Meier Plotter: the mRNA gene chip of “Start KM Plotter for breast cancer” module was used to analyze the prognostic value of HOXB3 mRNA expression in all breast cancers. - Breast Cancer Gene-Expression Miner (bc-GenExMiner) v4.3: we used the “analysis-correlation-targeted” module to get the total correlation heatmap of HOXB3 expression with clinicopathologic parameters. In addition, we used the “analysis-expression-targeted” module to find the relationship between HOXB3 and the clinicopathologic parameters of breast cancer. - Metascape: we used GEO2R to analyze the GSE27447 and get the top correlated gene lists with HOXB3 and submit them in the Metascape to get the gene enrichment analysis. - TIMER: we used the “Correlation” module to analyze the correlation between HOXB3 expression and the target genes, and we explored the association of immune infiltration levels among cancers with different SCNAs affecting HOXB3 expression in the “SCNA” module.
