# Supplementary figures and images for: Loss of HOXB3 correlates with the development of hormone receptor negative breast cancer
Source: PeerJ. 2020 Nov 20;8:e10421. doi: 10.7717/peerj.10421 (PMC7682434; doi:10.7717/peerj.10421)

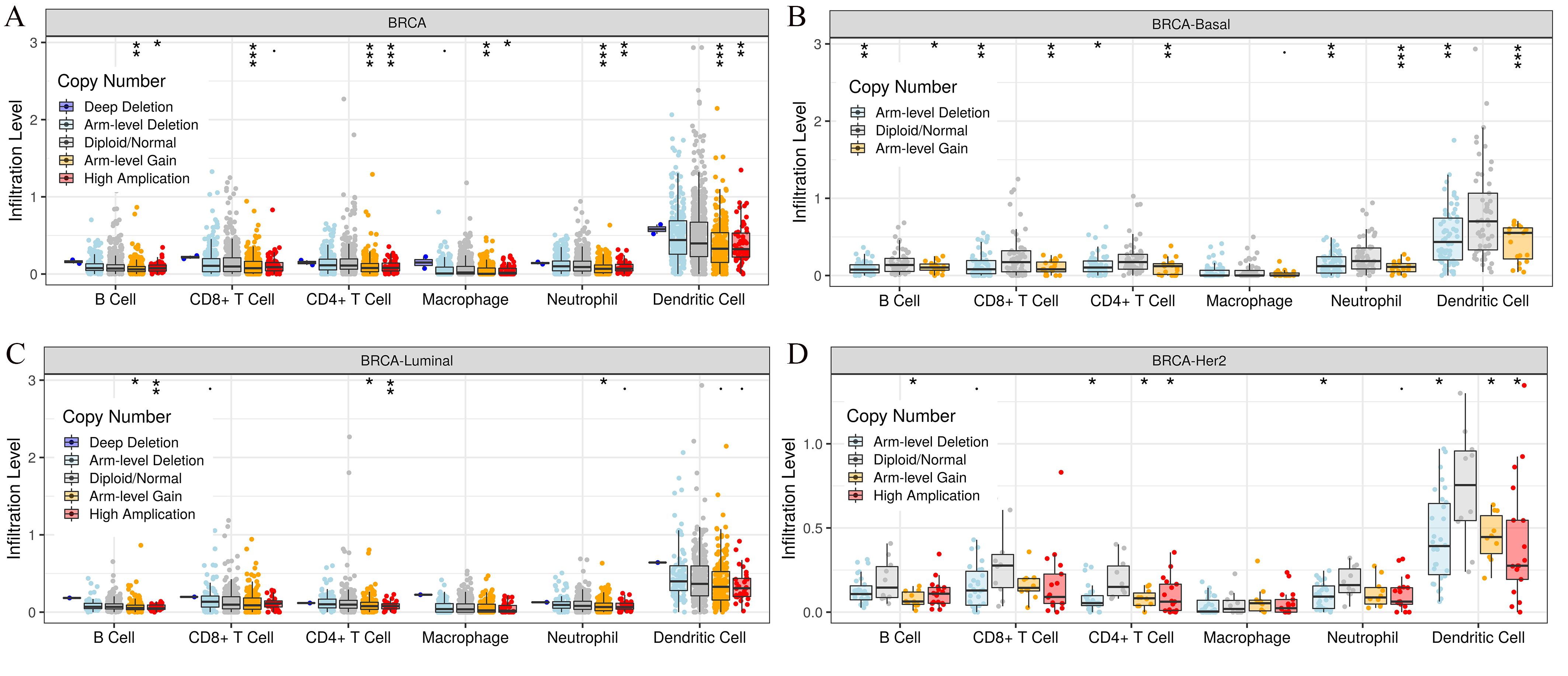

Supplement: Supplemental Information 1 — (A) BRCA (breast invasive carcinoma), (B) BRCA-Basal (breast carcinoma-basal), (C) BRCA-Luminal (breast cancer-luminal), (D) BRCA-Her2 (breast cancer-her2). [file peerj-08-10421-s001.png]

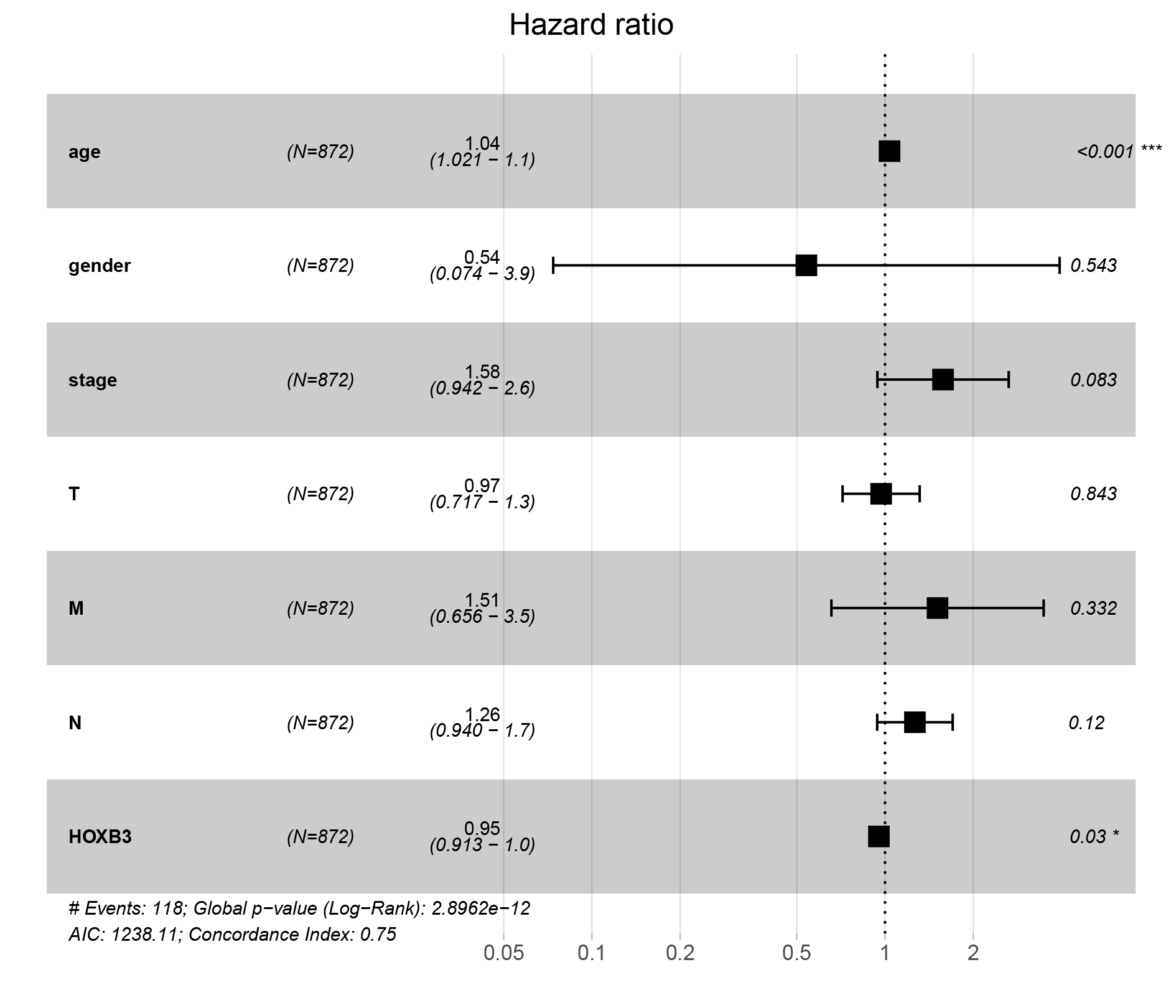

Supplement: Supplemental Information 2 [file peerj-08-10421-s002.png]

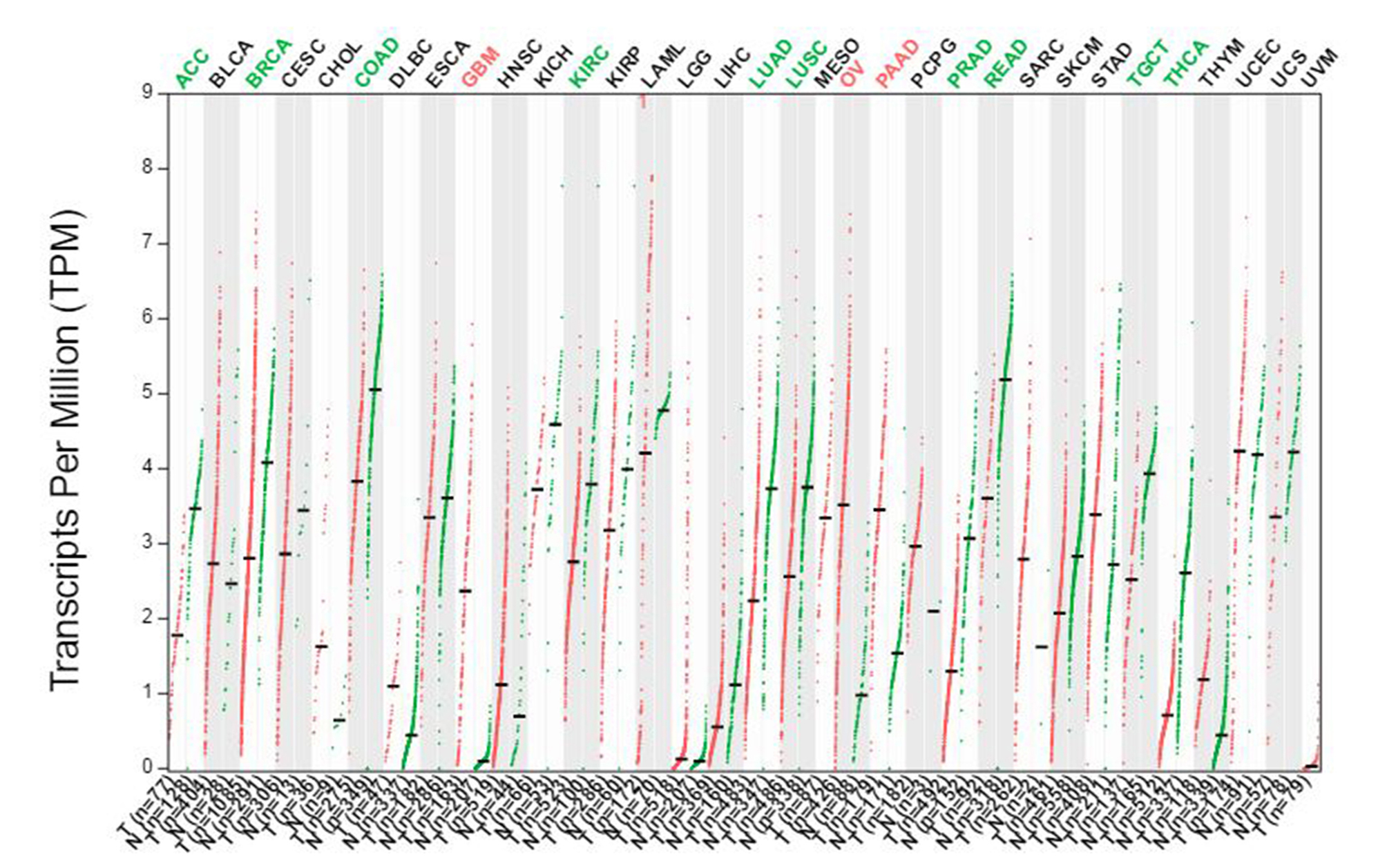

Supplement: Supplemental Information 3 [file peerj-08-10421-s003.png]

Figure 1C

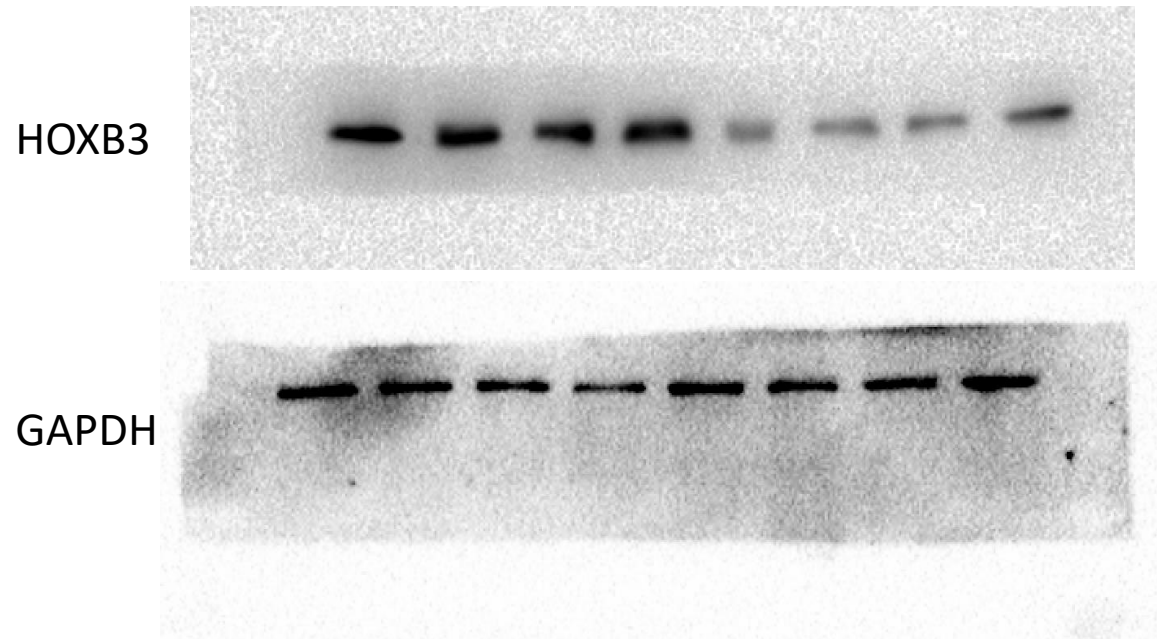

Figure 5C

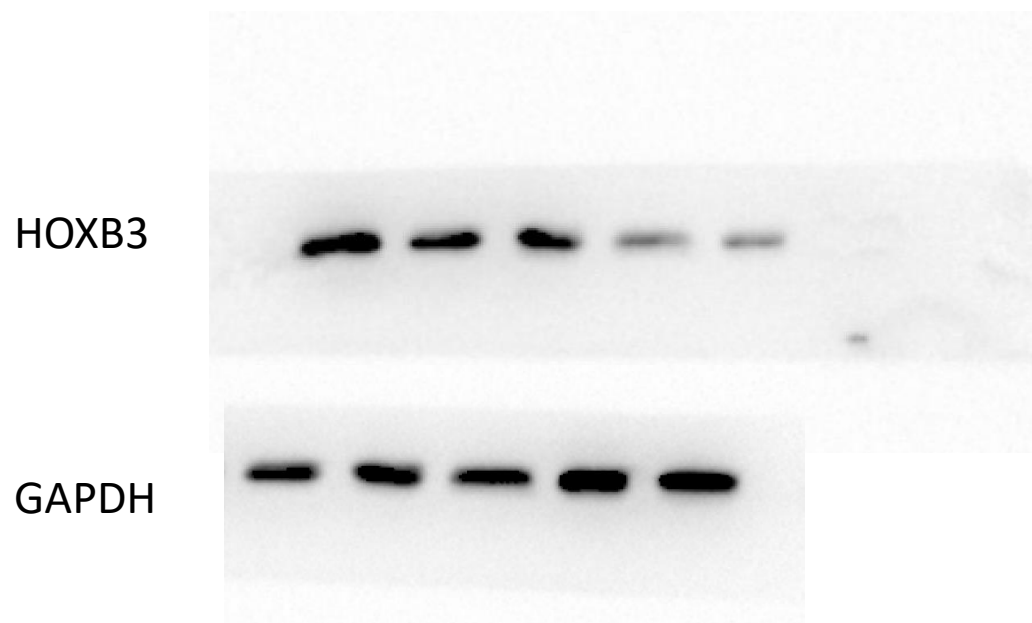

Figure 5D

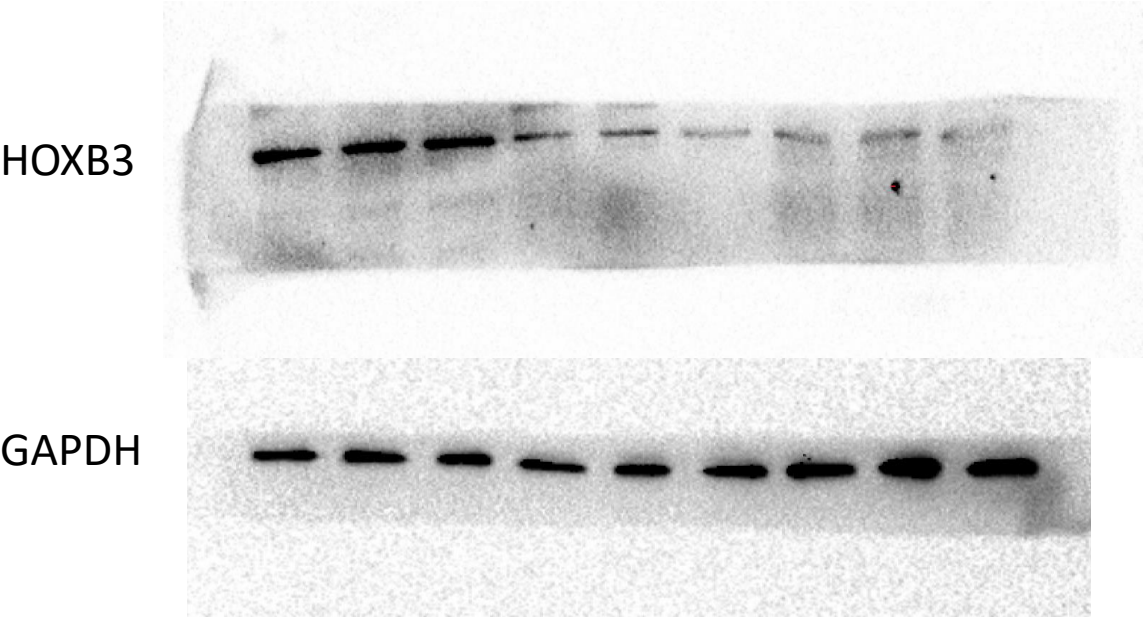

Supplement: Supplemental Information 7 [file peerj-08-10421-s007.pdf]
