# Supplementary material for: Loss of HOXB3 correlates with the development of hormone receptor negative breast cancer
Source: PeerJ. 2020 Nov 20;8:e10421. doi: 10.7717/peerj.10421 (PMC7682434; doi:10.7717/peerj.10421)
Supplement: Supplemental Information 4 — Kaplan-Meier Plotter auto select best cutoff to split patients (all possible cutoff values between the lower and upper quartiles are computed, and the best performing threshold is used as a threshold). Bold values indicated P¡0.05. N represents number of included patients. RFS, recurrence free survival; OS, overall survival; DMFS, distant metastasis free survival; PPS, post progression survival; HR, hazard ratio; CI, confidence interval. [file peerj-08-10421-s004.docx]

**Supplementary Table 1:**

**Survival analyses of HOXB3 in breast cancer.**

| **survival** | **breast cancer** | | | **triple-negative breast cancer** | | |
| --- | --- | --- | --- | --- | --- | --- |
|  | **N** | **HR (95%CI)** | **P** | **N** | **HR (95%CI)** | **P** |
| **RFS 208414_s_at**  **228904_at** | 1764 | 0.80 (0.72-0.89)  0.69 (0.59-0.81) | **6e-05**  **4.1e-06** | 161 | 1.38 (0.88-2.17)  1.34 (0.75-2.40) | 0.16  0.32 |
| **OS 208414_s_at**  **228904_at** | 626 | 0.83 (0.66-1.05)  0.66 (0.47-0.94) | 0.13  **0.02** | 0 | /  / | /  / |
| **DMFS 208414_s_at**  **228904_at** | 664 | 1.14 (0.94-1.38)  1.25 (0.90-1.74) | 0.19  0.18 | 28 | 3.18 (0.76-13.34)  8.47 (0.94-75.88) | 0.095  **0.022** |
| **PPS 208414_s_at**  **228904_at** | 173 | 0.81 (0.61-1.05)  1.35 (0.94-1.94) | 0.11  0.11 | 0 | /  / | /  / |

Kaplan-Meier Plotter auto select best cutoff to split patients (all possible cutoff values between the lower and upper quartiles are computed, and the best performing threshold is used as a threshold). Bold values indicated P<0.05. N represents number of included patients. RFS, recurrence free survival; OS, overall survival; DMFS, distant metastasis free survival; PPS, post progression survival; HR, hazard ratio; CI, confidence interval.
