# Supplementary material for: Loss of HOXB3 correlates with the development of hormone receptor negative breast cancer
Source: PeerJ. 2020 Nov 20;8:e10421. doi: 10.7717/peerj.10421 (PMC7682434; doi:10.7717/peerj.10421)
Supplement: Supplemental Information 5 — Bold values indicated P¡0.05. HR, hazard ratio; CI, confidence interval. [file peerj-08-10421-s005.docx]

**Supplementary Table 2:**

**Univariate analysis and multivariate analysis of the correlation of HOXB3 expression with OS among breast cancer patients.**

| **Parameter** | **Univariate analysis** | | | **Multivariate analysis** | | |
| --- | --- | --- | --- | --- | --- | --- |
|  | **HR** | **95%CI** | **P** | **HR** | **95%CI** | **P** |
| **age** | 1.03 | 1.02-1.05 | **<0.001** | 1.04 | 1.02-1.05 | **<0.001** |
| **gender** | 0.89 | 0.12-6.36 | 0.905 | 0.54 | 0.07-3.94 | 0.543 |
| **stage** | 2.11 | 1.66-2.69 | **<0.001** | 1.58 | 0.94-2.64 | 0.083 |
| **T** | 1.46 | 1.17-1.82 | **0.001** | 0.97 | 0.72-1.31 | 0.843 |
| **M** | 6.52 | 3.65-11.65 | **<0.001** | 1.51 | 0.66-3.48 | 0.332 |
| **N** | 1.70 | 1.41-2.06 | **<0.001** | 1.26 | 0.94-1.70 | 0.120 |
| **HOXB3** | 0.87 | 0.74-1.02 | 0.079 | 0.95 | 0.91-1.00 | **0.030** |

Bold values indicated P<0.05. HR, hazard ratio; CI, confidence interval.
